# Mitochondrial Transfer Between Cancer and T Cells: Implications for Immune Evasion

**DOI:** 10.3390/antiox14081008

**Published:** 2025-08-18

**Authors:** Soohyun Chun, Jin An, Man S. Kim

**Affiliations:** 1 Translational-Transdisciplinary Research Center, Medical Science Research Institute, Kyung Hee University Hospital at Gangdong, College of Medicine, Kyung Hee University, Seoul 05278, Republic of Korea; soohyunchun22@khu.ac.kr; 2 Department of Medicine, College of Medicine, Kyung Hee University, Seoul 02447, Republic of Korea; 3 Department of Pulmonary, Allergy and Critical Care Medicine, Kyung Hee University Hospital at Gangdong, College of Medicine, Kyung Hee University, Seoul 05278, Republic of Korea; anjin7487@khu.ac.kr

**Keywords:** mitochondrial transfer, oxidative stress, tumor microenvironment, T cell exhaustion, immune evasion, MERCI methodology, single-cell analysis, tunneling nanotubes, immunotherapy, cancer metabolism

## Abstract

Intercellular mitochondrial transfer in the tumor microenvironment (TME) is a paradigm-shifting process that redefines cancer–T cell crosstalk. This review explores its dual nature as both a tumor immune evasion strategy and a promising therapeutic avenue. Crucially, oxidative stress acts as a key regulator, inducing tunneling nanotube (TNT) formation to facilitate this organelle exchange. Tumors exploit this by transferring dysfunctional, reactive oxygen species (ROS) generating mitochondria to T cells to induce senescence while simultaneously hijacking healthy mitochondria from T cells to empower their own metabolism. This directional exchange, quantified by computational tools like mitochondrial-enabled reconstruction of cellular interactions (MERCI), is linked to poor clinical outcomes. Transfer occurs via TNTs, extracellular vesicles, and direct contact. Conversely, the therapeutic transfer of healthy mitochondria from sources like mesenchymal stromal cells can revitalize exhausted T cells, improving chimeric antigen receptor T (CAR-T) cell efficacy. Clinical translation is guided by emerging biomarkers, including circulating mitochondrial DNA (mtDNA), mitochondrial haplogroups, and the tumor mitochondrial transfer (TMT) score. Harnessing this biological axis for next-generation immunotherapies requires overcoming challenges in transfer efficiency and standardization to effectively modulate the tumor redox landscape and immune response.

## 1. Introduction

Beyond their classic role as metabolic powerhouses, mitochondria are now recognized as critical mediators in the intricate interplay between cancer cells and T cells, where metabolic reprogramming dictates therapeutic success [1,2]. A recent paradigm shift in understanding intercellular interactions is the discovery of the direct transfer of mitochondria between cells within the tumor microenvironment (TME). This phenomenon challenges traditional concepts of cellular autonomy, revealing new dimensions of cancer–immune cell crosstalk.

Central to these interactions is the role of oxidative stress as both a consequence and driver of mitochondrial transfer. Reactive oxygen species (ROS), primarily generated through mitochondrial respiration and environmental stress, create a complex redox landscape that fundamentally influences organelle trafficking dynamics. Clinical evidence from lung cancer studies demonstrates that oxidative stress and antioxidant resistance play crucial roles in tumor metastasis, with mitochondrial dysfunction caused by mtDNA damage serving as a key molecular event [3]. Notably, oxidative stress induces the formation of tunneling nanotubes via Cofilin regulation [4], creating direct physical conduits for mitochondrial exchange between cancer and immune cells.

The traditional framework of cancer immune evasion extends beyond well-characterized mechanisms to a more insidious strategy: the transfer of dysfunctional mitochondria to infiltrating T cells. Clinical specimens have revealed that mitochondrial DNA (mtDNA) mutations in tumor-infiltrating lymphocytes (TILs) are frequently shared with cancer cells, indicating a direct mitochondrial transfer from malignant cells to immune effectors [5]. These transferred mitochondria can resist mitophagy, impairing T cell metabolic function and senescence. The mitochondrial-enabled reconstruction of cellular interactions (MERCIs) computational framework systematically tracks this process, revealing a near-unidirectional transfer from T cells to cancer cells that “metabolically empowers” cancer cells while “depleting immune cells”. This mitochondrial “hijacking” is linked to poor clinical outcomes across different cancer types [6].

The mechanistic diversity of mitochondrial transfer encompasses tunneling nanotubes (TNTs), extracellular vesicles (EVs), and direct cell–cell contacts. Conversely, this process can be harnessed for therapeutic benefit. Recent investigations have established that bone marrow stromal cells can enhance T cell metabolic fitness through nanotube-mediated transfer of healthy mitochondria [7]. This duality—where transfer can either suppress immunity or enhance it—defines the central challenge and opportunity in this field. Although healthy mitochondrial transfer can revitalize exhausted T cells and enhance immunotherapy responses, the hijacking of mitochondria by cancer cells represents a novel mechanism of immune suppression [8]. The development of sophisticated computational methods is crucial for dissecting these opposing outcomes and their underlying mechanisms [9,10].

**Figure 1:**
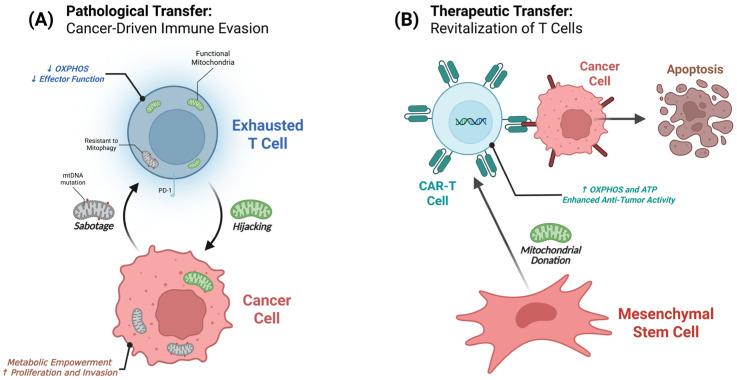
The dual role of intercellular mitochondrial transfer in the tumor microenvironment. This figure illustrates the opposing outcomes of mitochondrial transfer between cancer cells and T cells. (**A**) In a pathological context, cancer cells orchestrate immune evasion by hijacking functional mitochondria from T cells and, in turn, transferring dysfunctional mitochondria to induce T cell exhaustion. (**B**) In a therapeutic context, healthy donor cells, such as mesenchymal stromal cells (MSCs), can transfer functional mitochondria to exhausted T cells, leading to their metabolic revitalization and enhanced antitumor activity. Key mechanisms include tunneling nanotubes (TNTs) and extracellular vesicles (EVs). PD-1, Programmed cell death protein 1. Figure created in BioRender. mol, C. (2025). https://BioRender.com/z4rn9nb. The up and down arrows indicate increases and decreases, respectively.

This review synthesizes the current understanding of mitochondrial transfer between cancer and T cells, examining its diverse mechanisms, dual-edged functional consequences, and emerging therapeutic strategies. By addressing both immune evasion and therapeutic enhancement, this work provides a roadmap for next-generation immunotherapies targeting this fundamental aspect of cancer–immune interaction, which may help overcome the limitations of current treatments [11,12]. This dual nature of mitochondrial transfer—as both an immune evasion mechanism and a therapeutic opportunity—is illustrated in Figure 1, which depicts the contrasting outcomes depending on the cellular context and directionality of organelle exchange.

## 2. Mitochondrial Transfer Mechanisms in the TME

### 2.1. TNTs: Primary Conduits for Mitochondrial Exchange

TNTs are F-actin-based, membrane-bound structures that create continuous cytoplasmic channels, facilitating bidirectional organelle exchange and enabling cancer cells to exploit “mitochondrial hijacking” to reshape immune cell metabolism. TNTs exhibit remarkable structural complexity and therapeutic potential. For instance, they can function as natural biophotonic conveyors, where near-infrared light can direct organelle transport with high precision, offering a potential therapeutic strategy to inhibit mitochondrial hijacking from immune to cancer cells [9].

The formation of TNTs involves complex cytoskeletal reorganization, often driven by microenvironmental stress. Notably, oxidative stress, a key feature of the TME, induces TNT formation via the regulator Cofilin. Cofilin, an actin-depolymerizing factor, must be precisely regulated, as its local inactivation is thought to be necessary to allow for the stable actin polymerization required for TNT elongation and persistence. The inhibition of Cofilin suppresses this process and promotes senescent tumor cell death, highlighting its therapeutic potential [4]. Developmental pathways, such as Wnt signaling, also modulate TNT dynamics and cytoskeletal remodeling. Both canonical (β-catenin-dependent) and non-canonical pathways are implicated in regulating the cytoskeletal architecture necessary for TNT formation, suggesting that cancer cells may co-opt these pathways to enhance mitochondrial manipulation [13].

TNT-mediated transfer exhibits diverse functional outcomes, with distinct mechanisms documented in different cancer contexts. In adenoid cystic carcinoma, TNTs facilitate complete cell fusion, rescuing metabolic dysfunction and boosting malignancy [14]. This mechanism appears to be an exploitation of an evolutionarily conserved rescue process, as seen in human neural stem cells that use TNTs to rescue ischemic neurons [15]. In prostate cancer, TNTs work synergistically with EVs to create a more robust intercellular communication network, enhancing tumor cell survival and coordinating resistance mechanisms across the tumor population [16]. Consequently, targeting TNT-mediated mitochondrial transfer has emerged as a promising strategy for cancer therapy [17]. While these findings suggest potential cancer-type-specific variations in TNT function, additional studies across multiple cancer types are needed to establish whether these represent consistent mechanistic patterns or context-dependent processes that may occur in various tumor contexts.

### 2.2. EV-Mediated Mitochondrial Transfer

EVs represent another pathway enabling long-range organelle trafficking in the TME. This mechanism involves the packaging of intact mitochondria or their components within membrane-bound vesicles. The biogenesis of these so-called mitochondria-containing extracellular vesicles (mitoEVs) is a complex process, thought to involve the outward budding of the plasma membrane to enclose cytoplasmic cargo. This cargo is not random and can include specific mitochondrial proteins, metabolites, and mtDNA, which can subsequently alter the metabolic state of distant recipient cells [18]. Due to their tissue-specific composition, mitoEVs also hold promise as biomarkers for disease monitoring [18]. The therapeutic potential of EV-mediated mitochondrial transfer has been explored in various contexts, including cerebrovascular diseases, suggesting that engineered EVs could restore mitochondrial function in exhausted T cells by replenishing their OXPHOS capacity, thereby enhancing their persistence and effector functions in cancer immunotherapy [19]. A key molecular mechanism governing this process involves the Purinergic Receptor P2X 7 (P2X7) receptor. This ATP-gated ion channel, upon sustained activation, forms a large non-selective pore, a process linked to the shedding of membrane vesicles, including those containing mitochondria. This demonstrates that purinergic signaling can control this exchange in immune cells like microglia [20].

### 2.3. Direct Cell–Cell Contact and Transfer Regulation

Mitochondrial transfer also occurs through physical membrane connections facilitated by specialized protein machinery. For example, Connexin 43 is a critical regulator, forming gap junction channels that permit mitochondrial passage, particularly when recipient cells are under oxidative stress [21]. These channels are formed when a Connexin hemichannel (connexon) on one cell docks with its counterpart on an adjacent cell, creating a direct cytoplasmic conduit for small molecules and, remarkably, entire organelles. The truncated isoform of Connexin 43, GJA1-20k, also participates in this process, suggesting multiple layers of regulation. Adaptor proteins like Miro1 further regulate the process by controlling mitochondrial mobility and selection for transfer. As a Rho-GTPase, Miro1 anchors mitochondria to motor proteins like kinesin, which move along microtubule tracks, and is crucial for docking mitochondria at the site of intercellular transfer, with different cancer types such as melanoma employing distinct molecular machinery [22].

Mesenchymal stem cells (MSCs) are prominent mediators of this direct transfer, exemplifying the dual therapeutic and pathological potential of mitochondrial exchange. MSCs can enhance T cell metabolic fitness through beneficial mitochondrial transfer [7], while carcinoma-associated MSCs can exploit the same mechanism to promote ovarian cancer heterogeneity and metastasis, enhancing cancer stemness and chemoresistance [23]. Alternatively, enhancing intrinsic mitochondrial function through deletion of negative regulators such as methylation-controlled J protein (MCJ) can significantly improve CD8+ T cell adoptive therapy efficacy by increasing mitochondrial respiration and ATP production [24].

**Figure 2:**
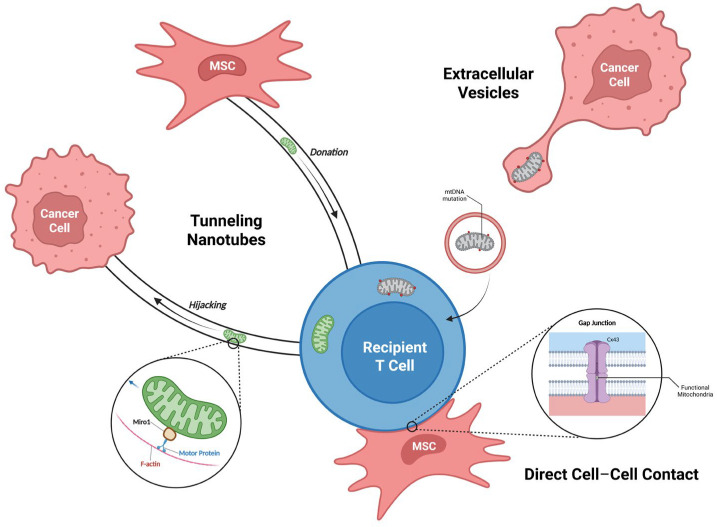
Key mechanisms and molecular regulators of intercellular mitochondrial transfer. This figure illustrates the three primary pathways for mitochondrial transfer to a central recipient T cell: (1) tunneling nanotubes (TNTs), (2) extracellular vesicles (EVs), and (3) direct cell–cell contact via gap junctions. (1) TNTs are F-actin-based tubes that form direct cytoplasmic bridges. The transport of mitochondria along these actin tracks is an active process facilitated by motor proteins, with the adaptor protein Miro1 anchoring the mitochondria to the motor complex. The stability of the actin tracks requires the local inactivation of actin-depolymerizing factors like Cofilin. (2) EV-mediated transfer involves the packaging of mitochondria or their components into vesicles. The release of these EVs can be triggered by high concentrations of extracellular ATP, which activates the P2X7 receptor on the donor cell. (3) Direct transfer can occur through gap junction channels, composed of Connexin 43 (Cx43) proteins, which allow for the passage of organelles between adjacent cells, such as from a mesenchymal stromal cell (MSC) to a T cell. Each pathway contributes to the complex intercellular communication within the tumor microenvironment. Figure created in BioRender. mol, C. (2025). https://BioRender.com/go4stwh.

This contextual duality, which is explored further in Section 6, underscores the complexity of targeting this pathway. The broader therapeutic implications are significant, with horizontal mitochondrial transfer explored as a novel bioenergetic tool in regenerative medicine and for treating various metabolic and neurodegenerative diseases [25,26]. The three primary mechanisms of intercellular mitochondrial transfer and their key molecular regulators are illustrated in Figure 2.

### 2.4. Detection and Quantification of Mitochondrial Transfer

As shown in Figure 2, the primary pathways for mitochondrial transfer involve distinct molecular machinery and regulatory mechanisms. Quantifying mitochondrial transfer events at single-cell resolution is critical for understanding their clinical relevance. The MERCI computational framework was developed to trace mitochondrial trafficking between cancer and T cells using single-cell RNA sequencing data [6]. By analyzing mitochondrial gene expression, MERCI can identify recipient cells and has led to the development of the tumor mitochondrial transfer (TMT) score, a 17-gene signature that correlates mitochondrial transfer activity with poor patient survival in large cancer cohorts [27]. Complementing these computational approaches, advanced imaging technologies such as fast fluorescence lifetime imaging microscopy (FLIM) combined with stimulated emission depletion (STED) nanoscopy allow for direct, real-time visualization of mitochondrial movement through TNTs at nanoscale resolution [28]. The comparative features and relative contributions of these three primary transfer mechanisms are summarized in Table 1, highlighting their distinct molecular machinery, functional characteristics, and therapeutic targeting opportunities.

### 2.5. Key Regulatory Proteins and Cell-Type-Specific Expression Patterns

The directionality and efficiency of mitochondrial transfer are governed by differential expression of key regulatory proteins across TME cell types. The TMT score, comprising 17 genes implicated in nanotube formation and cytoskeletal remodeling, demonstrates strong correlation with tumor proliferation and poor survival across cancer types [6,27].

Critical regulatory proteins exhibit distinct cell-type expression patterns that determine transfer outcomes. Connexin 43 (CX43) in mesenchymal stromal cells forms gap junction channels enabling direct mitochondrial passage, particularly under oxidative stress [21]. In contrast, the P2X7 receptor in immune cells controls EV-mediated transfer through ATP-gated pore formation, providing precise regulation of mitochondrial exchange in microglia [20]. Miro1 serves as a universal adaptor protein, anchoring mitochondria to motor proteins and controlling transfer site docking, with cancer-type specific machinery variations observed in melanoma [22].

Cofilin regulation exemplifies the therapeutic potential of targeting these proteins. Oxidative stress-induced Cofilin inactivation enables TNT formation and pathological transfer, while Cofilin pathway inhibition suppresses this process and promotes tumor cell senescence [4]. The cell-type specific expression profiles of these regulatory proteins offer opportunities for biomarker development and precision therapeutic targeting, where the same molecular machinery can yield opposing outcomes depending on donor–recipient cell combinations.

The specificity and directionality of mitochondrial transfer are governed by multiple molecular determinants that operate at different cellular levels. Metabolic stress and energy demands in recipient cells serve as primary drivers of transfer directionality, with oxidatively stressed T cells preferentially receiving mitochondria through CX43-mediated channels [21]. The adaptor protein Miro1 provides selectivity by controlling which mitochondria are mobilized for transfer, anchoring specific organelles to motor proteins based on their functional status [22].

Transfer specificity is further regulated by cell surface recognition mechanisms and microenvironmental cues. The formation of TNTs via Cofilin-dependent pathways occurs preferentially between cells under shared stress conditions, creating selective intercellular bridges [4]. Additionally, the P2X7 receptor responds to local ATP concentrations, enabling context-dependent EV release that targets mitochondria to specific recipient cell populations [20]. This multi-layered control system ensures that mitochondrial transfer occurs in response to cellular need rather than randomly, explaining how the same molecular machinery can produce opposing therapeutic or pathological outcomes depending on the cellular context and metabolic state of donor–recipient pairs.

## 3. Mitochondrial Dysfunction and T Cell Exhaustion

### 3.1. mtDNA Integrity, Redox Stress, and T Cell Metabolic Impairment

Mitochondrial DNA (mtDNA) integrity is fundamental to T cell metabolic fitness and immune competence. In the TME, this integrity is compromised. Clinical specimens show that mtDNA mutations accumulate in tumor-infiltrating lymphocytes (TILs), often shared with cancer cells, suggesting the active transfer of defective mitochondria from tumors to T cells [5]. The accumulation of this damaged mtDNA impairs oxidative phosphorylation (OXPHOS) and forces a metabolic shift toward less efficient glycolysis, ultimately compromising long-term T cell functionality.

The molecular basis for this mtDNA damage is tightly linked to redox imbalance. Sustained T cell receptor activation, a constant feature in the TME, triggers excessive production of ROS, which directly inflicts oxidative damage on mtDNA. Genes encoding OXPHOS complexes I and III are preferentially affected, crippling the electron transport chain and creating a metabolic bottleneck that limits T cell proliferative capacity [31]. In response, T cells deploy adaptive antioxidant systems; the redox sensor Kelch-like ECH-associated protein 1 (KEAP1) is crucial for this, as persistent antigen stimulation primes chromatin for regulation via the KEAP1–nuclear factor erythroid 2-related factor 2 (NRF2) pathway, linking redox homeostasis directly to transcriptional control [32]. This delicate balance can be disrupted by external factors, such as the antibiotic tigecycline, which inhibits mitochondrial protein synthesis [33]. Furthermore, mitochondrial quality control via PTEN-induced kinase 1-mediated mitophagy is often impaired in exhausted T cells. This failure to clear damaged organelles leads to the accumulation of depolarized mitochondria, creating a feed-forward cycle of dysfunction that accelerates the functional decline of T cells [34].

The broader cellular antioxidant network extends beyond the KEAP1-NRF2 axis to encompass multiple defense mechanisms that become compromised during mitochondrial transfer. Cancer-associated fibroblasts (CAFs) demonstrate enhanced mitophagy and increased mtDNA release when exposed to oxidative stress, with ROS further promoting this pathological process [3]. This creates a feed-forward cycle where oxidative stress both drives mitochondrial transfer initiation and results from its consequences, particularly when dysfunctional organelles are transferred to immune cells.

### 3.2. The Role of Mitochondrial Transfer in Driving T Cell Dysfunction

The dysfunction described above is not solely cell-intrinsic; it is actively induced and exacerbated by intercellular mitochondrial transfer. This phenomenon involves a bidirectional exchange that dictates T cell fate. While the transfer of healthy mitochondria from stromal cells can be beneficial, the transfer of dysfunctional organelles from cancer cells promotes immune suppression [7]. Recent single-cell analyses using the MERCI framework have dissected this heterogeneity, classifying T cells by their mitochondrial transfer status [6]. This reveals that some T cells become “metabolically depleted” as their mitochondria are hijacked by tumor cells, while others may be “empowered” by receiving healthy mitochondria from other sources within the TME. This process, occurring via TNTs, EVs, or direct contact, fundamentally alters immune cell fate beyond simple metabolic support, acting as a master regulator of immune responses [7,35].

Cancer cells employ sophisticated mechanisms to achieve selective mitochondrial exchange that maximizes their metabolic advantage while compromising immune function. This selectivity operates through molecular control systems that distinguish between healthy and dysfunctional organelles during transfer events. Cancer cells demonstrate selective mitochondrial exchange with T cells as revealed by MERCI analysis, effectively “hijacking” mitochondria with intact OXPHOS machinery while transferring dysfunctional organelles [6], a dual strategy that is conceptually illustrated in Figure 1A and mechanistically in Figure 2. Simultaneously, these cells selectively export damaged, ROS-generating mitochondria through EV-mediated pathways, targeting dysfunctional organelles to immune cells where they resist mitophagy and accumulate to impair T cell metabolic function [5].

This asymmetric exchange is reinforced by the differential expression of transfer machinery components. Cancer cells upregulate elements of the TMT signature that facilitate mitochondrial acquisition while maintaining mechanisms that promote the export of mtDNA-damaged organelles to surrounding immune cells. The metabolic sensing mechanisms underlying this selectivity identify high-functioning organelles based on membrane potential and respiratory capacity, ensuring that cancer cells acquire the most metabolically beneficial mitochondria while disposing of their damaged counterparts. This sophisticated adaptation results in metabolically empowered cancer cells coupled with immunologically compromised T cells, representing a dual strategy that simultaneously enhances tumor fitness and suppresses antitumor immunity [6].

### 3.3. Phenotypic Hallmarks of T Cell Exhaustion

OXPHOS dysfunction is not merely a consequence but a causative trigger of T cell exhaustion. Mitochondrial insufficiency initiates the functional exhaustion of T cells through cell-intrinsic mechanisms, with exhausted T cells exhibiting diminished mitochondrial membrane potential, reduced expression of key OXPHOS complex subunits, and striking morphological abnormalities, including fragmented mitochondrial networks with shortened cristae [31]. This forces a reliance on glycolysis, which is insufficient in the nutrient-poor TME, leading to chronic energy deficits that compromise effector functions. This metabolic impairment is mechanistically and bidirectionally linked to the expression of inhibitory receptors like PD-1, TIM-3, and LAG-3; impaired metabolism promotes their expression, while signaling through these receptors, in turn, further suppresses mitochondrial function, creating a vicious cycle of exhaustion.

Different T cell subsets, however, show varying resilience. Tc17 cells and CD38-overexpressing CD8+ T cells display enhanced resistance to OXPHOS dysfunction due to distinct metabolic programming [36,37]. Similarly, tissue-resident memory (TRM) T cells have unique adaptations, such as reliance on the mevalonate-cholesterol pathway to synthesize Coenzyme Q, that support sustained antitumor function [2]. This heterogeneity offers insights into preserving T cell function. In contrast, CAR T cells are particularly vulnerable to mitochondrial dysfunction during ex vivo expansion and upon transfer into the hostile TME. The transition from precursor to terminally exhausted T cells through HIF-1α-mediated glycolytic reprogramming [31] limits their persistence and efficacy, especially in solid tumors, suggesting that metabolic engineering strategies could enhance CAR T cell functionality.

### 3.4. Therapeutic Strategies to Restore Mitochondrial Function

Several strategies aim to reverse T cell exhaustion by restoring mitochondrial function. These approaches target various aspects of mitochondrial biology, from enhancing biogenesis to improving quality control.

One approach involves metabolic supplementation and drug repurposing. Supplementation with nicotinamide riboside (NR), a precursor to the critical mitochondrial cofactor NAD+, enhances mitochondrial fitness, reduces ROS production, and promotes cytokine secretion in exhausted T cells [38]. Pharmacological inhibition of metabolic enzymes, such as prolyl 4-hydroxylase α1 (P4HA1), has also been shown to boost CD8+ T cell antitumor immunity by improving mitochondrial fitness [39]. Similarly, the repurposed drug Bezafibrate, a peroxisome proliferator-activated receptor (PPAR) agonist, promotes CD8+ T cell infiltration and enhances mitochondrial function in lung cancer models [40].

A second class of strategies uses genetic engineering and advanced delivery systems. T cells engineered to express myoglobin exhibit improved metabolism and superior antitumor activity, with mitochondrial morphology indicative of enhanced OXPHOS, such as increased cristae length [41]. Nanotechnology offers precise interventions, such as hydrogel-based “nanofactories” that co-deliver CAR T cells with mitophagy agonists to clear damaged mitochondria and drive T stem cell-like memory formation [42] or with STING-activating agents to relieve exhaustion by regulating mitochondrial dysfunction [43]. These advanced therapies can be tailored to specific disease contexts, such as mitigating T cell dysfunction in chronic lymphocytic leukemia (CLL) [44] or enhancing CAR T activity via co-expression of C-X-C motif chemokine ligand 13 (CXCL13) to potentiate PD-1 blockade responses [45].

The success of these therapies critically depends on timing and combination strategies. Combining mitochondrial restoration with other immunotherapies, such as checkpoint inhibition, can achieve synergistic effects. Ultimately, the development of robust biomarkers to assess mitochondrial function in patient T cells will be crucial for personalizing these promising therapeutic approaches.

## 4. Metabolic Rewiring in Cancer–Immune Cell Interactions

The metabolic landscape of the TME is a dynamic battlefield where the biochemical crosstalk between cancer and immune cells dictates therapeutic outcomes. Specific metabolic intermediates function as crucial signaling molecules that orchestrate these interactions. The emergence of mitochondrial transfer further complicates this landscape, creating new paradigms for understanding how metabolic communication shapes cancer progression [5].

### 4.1. Electron Transport Chain Modifications and Tumor Immunogenicity

The mitochondrial electron transport chain is a critical nexus where metabolic alterations directly influence tumor immunogenicity. Groundbreaking research has shown that selectively manipulating electron flow can dramatically enhance tumor susceptibility to immune-mediated destruction, independent of conventional interferon signaling [8]. These findings challenge the traditional view of mitochondrial function, revealing that subtle changes in electron transport can trigger profound immunological consequences.

Specifically, Complex II (succinate dehydrogenase, SDH) deficiency, but not Complex I loss, significantly reduces melanoma growth by enhancing antigen presentation. This effect stems from the intracellular accumulation of the metabolite succinate, which subsequently drives transcriptional and epigenetic activation of MHC-antigen processing genes. Succinate inhibits lysine-specific demethylases (KDMs), leading to increased histone methylation (e.g., H3K4me3) at the promoter regions of genes involved in antigen presentation, thereby increasing their expression. This succinate-driven pathway effectively reverses a key immune evasion strategy of cancer: the downregulation of the antigen presentation machinery [8]. The therapeutic feasibility of this approach has been demonstrated through strategies that rewire the electron transport chain to achieve potent antitumor responses without the side effects of broad respiratory suppression [8].

TRM CD8+ T cells also employ specialized metabolic programs to promote sustained tumor immunity. Their reliance on non-steroidal products of the mevalonate/cholesterol pathway, such as Coenzyme Q, supports mitochondrial respiration and memory formation, enhancing antitumor immunity [2].

### 4.2. Metabolite-Mediated Immune Signaling and Cell Communication

The TME generates a complex milieu of metabolites that function as signaling molecules. Lactate, for instance, profoundly shapes immune cell behavior through multiple mechanisms beyond its role as a metabolic waste product. Lactate accumulation creates distinct signaling environments that contribute to immune evasion, partly through lactylation, a post-translational modification that epigenetically reprograms cellular functions to generally favor immune suppression [46]. Dendritic cells and macrophages exhibit markedly different responses to lactate; in dendritic cells, lactate primarily alters STAT3 and MAPK signaling, impairing their antigen presentation capacity, whereas, in macrophages, it influences STAT1 and GSK-3β pathways, affecting their polarization state [47]. Consequently, therapeutic targeting of lactate signaling, particularly through inhibition of the monocarboxylate transporter 1 (MCT1), can reinvigorate antitumor immunity by metabolically rewiring dendritic cells [48]. This highlights how intercellular metabolic crosstalk is a key regulator of antitumor immunity, representing a promising avenue for immunotherapy [49].

Other immunometabolites also play crucial roles. Itaconate, produced from the TCA cycle intermediate cis-aconitate, modulates host inflammatory and defense responses through diverse mechanisms, including alkylation of proteins like KEAP1 (leading to NRF2 activation) and inhibition of enzymes such as SDH. It also functions as an extracellular signaling molecule [50]. Succinate can influence macrophage polarization within the TME, suggesting that succinate-based interventions may reprogram the immune landscape of tumors [51]. These examples underscore the immense complexity and therapeutic potential of cancer immunometabolism [52].

### 4.3. Tricarboxylic Acid (TCA) Cycle Modulation and Immunotherapy Enhancement

The TCA cycle is a central determinant of immunotherapy effectiveness. Strategic inhibition of key TCA cycle enzymes, such as pyruvate dehydrogenase and oxoglutarate dehydrogenase, can improve the efficacy of anti-PD-1 immunotherapy by simultaneously modulating metabolic flux and immune recognition pathways. This intervention activates the AMPK–CREB–ATF3 signaling pathway, which reduces PD-L1 expression while promoting glycolysis in cancer cells, creating a more favorable environment for T cell-mediated tumor destruction [53].

Metabolic pathways can also function as regulatory “checkpoints”. The CD73–adenosine axis, for example, suppresses immune responses by producing immunosuppressive adenosine from extracellular ATP. Inhibition of CD73 with agents like AB680 can suppress glioblastoma growth by blocking purine metabolism while promoting beneficial P2RY12+ microglial activation [54]. The immunometabolite L-2-hydroxyglutarate (L-2-HG) has also emerged as a key regulator, capable of reversing T cell exhaustion. Exhausted T cells show reduced L-2-HG levels, and supplementation improves mitochondrial metabolism, reduces inhibitory epigenetic marks (H3K27me3) on key effector genes, and enhances memory T cell differentiation. This highlights the therapeutic potential of metabolite-based approaches for overcoming T cell dysfunction [55]. These findings demonstrate that the TME induces deleterious metabolic switches in immune cells, and understanding these mechanisms provides insights into how metabolic interventions can reverse this immunosuppression [56].

### 4.4. Therapeutic Strategies Through Metabolic Reprogramming

Translating these metabolic insights into therapies is a rapidly evolving frontier. Nanomaterial-based metabolic reprogramming strategies offer unprecedented precision for delivering metabolic modulators directly to the TME. These can include carriers for metabolic inhibitors, systems that generate ROS to induce immunogenic cell death, or platforms that regulate nutrient availability within the tumor [57]. Dietary interventions also show promise; for example, specific fatty acids (FAs) like elaidic acid can enhance antigen presentation by modulating cellular metabolism [58]. Furthermore, the development of MHC class II inducers operating through metabolic editing offers a novel approach to enhance immune recognition in tumors that have downregulated this machinery [59].

The challenges and opportunities in targeting metabolism are vast. Elevated lactate, for instance, contributes to PD-1/PD-L1 immunotherapy resistance, but this also reveals opportunities for combination therapies that target both lactate transport and immune checkpoints [60]. Advanced analytical techniques like spatiotemporal metabolomics are providing unprecedented insights into the evolution of metabolic landscapes, facilitating personalized therapeutic strategies [61]. Ultimately, a comprehensive approach that includes reprogramming of the tumor vasculature endothelium, which controls nutrient supply and immune infiltration, will be key to modifying the TME for therapeutic benefit [62].

## 5. The Dual Role of Mitochondrial Transfer: Therapeutic Enhancement and Pathological Evasion

Intercellular mitochondrial transfer exemplifies the complex duality inherent in biological processes, where the same mechanism can yield profoundly different outcomes depending on the cellular context, donor–recipient combination, and mitochondrial functional status. In cancer immunology, mitochondrial transfer operates as a double-edged sword: it can be harnessed to enhance immune function and support therapeutic interventions, or it can be exploited by tumors to facilitate immune evasion and promote progression [1]. This section explores both facets of this phenomenon, from therapeutic applications to pathological consequences, and discusses the factors that determine its ultimate outcome.

### 5.1. Beneficial Transfer as a Therapeutic Strategy

The therapeutic potential of mitochondrial transfer is most evident when healthy mitochondria are transferred from donor cells to compromised recipient cells, enhancing their metabolic fitness and function. This principle is being explored through various innovative strategies. Agents like selenium nanoparticles can be used to activate selenoproteins, which in turn enhance the therapeutic mitochondrial transfer capacity of bone marrow-derived mesenchymal stromal cells (BMSCs), demonstrating that the donor cell’s capacity can be pharmacologically augmented [63]. Beyond augmenting endogenous transfer, direct mitochondrial transplantation is emerging as a cell-free therapy. This approach, which involves isolating healthy mitochondria and delivering them to target tissues, has shown efficacy in preclinical models of mitochondrial disorders and is being explored for its regenerative potential [64].

The clinical relevance of these approaches is underscored by the development of sophisticated biomarkers. Circulating cell-free mtDNA (cf-mtDNA) has emerged as a valuable liquid biopsy tool. Its features, including copy number, specific mutations, and fragmentomics, can predict the response to transarterial chemoembolization in hepatocellular carcinoma [65] and guide adjuvant chemotherapy decisions in colorectal cancer patients with mismatch repair deficiency [66]. Inherited mitochondrial genetics, specifically mitochondrial haplogroup T, have also been identified as a novel, independent predictor of resistance to immune checkpoint inhibitors in melanoma patients [67]. At the protein level, proteomics of melanoma responses to immunotherapy have revealed mitochondrial-dependent signatures that correlate with treatment efficacy [68], while specific biomarkers like lysocardiolipin acyltransferase 1 (LCLAT1) show prognostic potential in hepatocellular carcinoma by reflecting tumor immunity status and mitochondrial function [69].

Advanced therapeutic technologies are also being developed in parallel. Targeting mitochondria-derived vesicles (MDVs)—specialized EVs that selectively encapsulate and transport damaged mitochondrial proteins under oxidative stress—represents a cutting-edge approach. These MDVs can be engineered for targeted delivery to either block detrimental communication or enhance beneficial transfer [29]. Modulating mitochondrial outer membrane proteins, such as voltage-dependent anion channel 2 (VDAC2), offers another strategy; its loss can elicit tumor destruction while simultaneously promoting a pro-inflammatory response [70]. For adoptive cell therapies, CAR-NK cells can be “armored” with agents like Neoleukin-2/15 to activate c-Myc/NRF1 pathways, enhancing their mitochondrial function and therapeutic efficacy in solid tumors [71].

The specificity of transfer can also be leveraged for precise immunomodulation. For instance, mitochondrial transfer from MSCs to allogeneic Tregs is HLA-dependent and improves their immunosuppressive activity, offering potential applications in controlling autoimmunity or transplant rejection [72]. In other contexts, such as acute ischemic stroke, umbilical MSCs can mitigate T cell compartment shifts and restore the Th17/Treg balance via mitochondrial transfer [73]. Similarly, enhancing the function of NK cells by modulating their mitochondrial dynamics through cytokine cocktails (e.g., IL-12/15/18) is another promising avenue [74]. Multiple strategies are being developed to enhance transfer specificity and efficiency for therapeutic applications. Optical manipulation using near-infrared light can direct organelle transport with high precision through TNTs, offering potential for selective inhibition of pathological transfer while promoting beneficial exchanges [9]. Pharmacological enhancement approaches include selenium nanoparticles that activate selenoproteins to augment the therapeutic mitochondrial transfer capacity of bone marrow-derived mesenchymal stromal cells [63]. Additionally, genetic modifications such as MCJ deletion can enhance intrinsic mitochondrial function and improve chimeric antigen receptor T (CAR-T) cell efficacy [24], while engineering T cells to express myoglobin improves their metabolic capacity and antitumor activity [41]. Clinical biomarkers for assessing mitochondrial transfer activity are summarized in Table 2, showing their performance metrics and validation status across different cancer types.

### 5.2. The Context of Mitochondrial Transfer: From Broad Biology to Specific Cell Types

The significance of mitochondrial transfer is rooted in its broad biological relevance, as highlighted by comprehensive reviews on the topic [75]. This horizontal exchange has been documented to influence the fate of various immune cells, including B lymphocytes, whose differentiation can be modulated by MSC-derived mitochondria [76]. The therapeutic promise has spurred the development of cell-free mitochondrial transplantation for conditions like osteoarthritis [77], and the general concept of targeting mitochondrial transfer is now considered a promising therapeutic strategy across many diseases [78]. A key example of its regenerative potential is in cartilage repair, where mitochondrial transfer from BMSCs protects chondrocytes against degenerative mitochondrial dysfunction [79].

### 5.3. Detrimental Transfer: A Mechanism of Immune Evasion and Tumor Progression

In stark contrast to its therapeutic potential, mitochondrial transfer is also a sophisticated mechanism hijacked by cancer cells to cripple the immune response and fuel their own survival and spread. CAFs transfer mitochondria to aggressive breast cancer cells, increasing their migratory capacity and altering their metabolic profile [80]. This movement of mitochondria, both within breast cancer cells and from the stroma, is now recognized as a critical factor in promoting aggressiveness and chemoresistance [81]. Furthermore, highly metastatic tumor cells can disseminate metastasis-enhancing mtDNA mutations to less aggressive cells via EVs, creating a field effect that promotes widespread invasiveness [82].

Cancer cells also co-opt other stromal cells in the TME. Adipose stem cells can serve as mitochondrial reservoirs for cancer cells, enhancing their metabolic capacity and survival potential [83]. In the central nervous system, brain tumor-initiating cells transfer mitochondria to neighboring astrocytes, corrupting their supportive function to facilitate tumor growth [84]. This phenomenon is not passive; environmental cues within the TME can actively redirect cellular fate decisions following mitochondrial transfer, altering normal trafficking patterns and promoting malignancy [85].

### 5.4. Challenges and Future of Mitochondrial Transfer Therapies

The clinical translation of these findings faces several key challenges. A central issue is the complex interplay between different therapeutic vesicles, such as EVs and isolated mitochondria, which needs further clarification for applications in regenerative medicine [86]. Optimizing transfer efficiency is another hurdle. While some cell populations, such as highly purified, rapidly expanding MSC clones (RECs), can be enriched for superior transfer capabilities, current overall rates can be low, posing a scalability challenge [87].

Understanding the intricate biology of mitochondrial transfer will continue to open new therapeutic avenues. This includes elucidating how CX43-mediated transfer maintains stemness in leukemia cells [30] or its role in preventing neuronal ferroptosis after spinal cord injury [88]. Finally, standardizing protocols for mitochondrial isolation, preservation, and delivery [89,90,91] and ensuring the safety and long-term effects of organelle-based therapies such as chimeric cell therapy [92] are critical for moving these innovative strategies from the bench to the bedside. Despite therapeutic promise, mitochondrial transfer therapies carry potential risks that require evaluation. As demonstrated with carcinoma-associated MSCs that exploit mitochondrial transfer to promote ovarian cancer heterogeneity and metastasis [23], therapeutic mitochondrial transfer could inadvertently enhance malignant cell fitness if cancer cells hijack therapeutically delivered organelles. The contextual duality observed with MSCs—where the same transfer mechanism can be either therapeutic or pathological depending on cellular context [23,72]—highlights the need for careful patient selection and delivery strategies to minimize off-target effects on residual cancer cells. Current therapeutic strategies targeting mitochondrial transfer are outlined in Table 3, categorized by their mechanisms of action and development stages.

## 6. Computational Methods and Single-Cell Technologies

The study of mitochondrial transfer has been revolutionized by computational methodologies and single-cell technologies that enable systematic analysis with unprecedented precision.

### 6.1. MERCI: A Computational Framework for Quantifying Transfer

MERCI represents a breakthrough in the field. MERCI is a statistical deconvolution method that leverages single-cell RNA sequencing to trace and quantify mitochondrial trafficking between cancer and T cells [6]. Its core principle involves identifying single cells whose transcriptome contains a “mixed signature” of mitochondrial DNA variants from two different cell origins (e.g., a T cell expressing some cancer-specific mtDNA mutations). The algorithm then statistically calculates the likely proportion of transferred mitochondria, thereby inferring the direction and relative extent of the transfer. Applying MERCI to skin and esophageal cancer samples revealed a reproducible cancer phenotype associated with mitochondrial transfer, characterized by the upregulation of genes linked to cytoskeleton remodeling and energy production [6].

This framework led to the creation of the tumor mitochondrial transfer (TMT) score, an integrated score based on 17 specific genes implicated in nanotube formation and transfer. Analysis of over 10,000 cancer samples demonstrated a strong correlation between a high TMT score and both rapid tumor proliferation and poor patient survival across multiple cancer types [6,27]. This provides a powerful tool for tracking cancer energy hijacking and suggests that targeting mitochondrial transfer could be a viable therapeutic strategy [27].

Despite these advances, quantifying mitochondrial transfer efficiency in clinical samples faces significant limitations. MERCI’s moderate sensitivity of 56.3% indicates that substantial transfer events may be missed, particularly low-level exchanges that could still be functionally important [6]. The computational approach relies on transcriptomic signatures that may not capture the dynamic nature of organelle trafficking, as RNA expression can lag behind actual mitochondrial movement. Additionally, current computational methods require high-quality single-cell RNA sequencing data, which may not be feasible in all clinical settings [6]. The heterogeneity of clinical samples and lack of standardized protocols across institutions further challenge reproducible quantification. The heterogeneity of clinical samples and lack of standardized protocols across institutions further challenge reproducible quantification in diverse clinical settings.

### 6.2. Single-Cell Analysis for Functional Interrogation

Single-cell technologies have been pivotal in functionally characterizing mitochondrial transfer. For instance, the isolation of highly purified, rapidly expanding clones (RECs) of MSCs—isolated using single-cell sorting for high expression of markers like CD90 and CD271—has demonstrated that specific subpopulations possess superior mitochondrial transfer efficiency compared to heterogeneous cultures. RECs exhibit higher mitochondrial content and membrane potential and more effectively restore mitochondrial function, including ATP production and OXPHOS capacity, in recipient cells [87]. Single-cell analyses have also provided critical insights into the molecular machinery of transfer, such as the role of Connexin 43 (CX43) gap junctions in mediating mitochondrial transfer from bone marrow stromal cells to leukemia stem cells to maintain their stemness through metabolic remodeling [30]. Similarly, in a spinal cord injury model, single-cell RNA sequencing revealed that MSCs attenuate neuronal ferroptosis. This analysis was crucial as it identified that neurons were the primary cell type undergoing ferroptosis and that MSCs specifically restored the neuronal mitochondrial pool via TNTs, a finding that would be obscured by bulk analysis [88].

### 6.3. Advanced Imaging and Real-Time Tracking

Advanced imaging has provided direct visual evidence of organelle trafficking. Fluorescent protein tagging enables precise mitochondrial labeling for continuous monitoring with minimal cytotoxicity [89]. Using dual-fluorescence labeling (e.g., mito-GFP and mito-RFP), researchers can simultaneously track bidirectional mitochondrial transfer between different cell types, as demonstrated in co-cultures of MSCs and retinal pigment epithelial cells, where transfer via TNTs was directly observed [90]. Real-time, high-content imaging systems have captured the entire process of exogenous mitochondrial endocytosis and integration into recipient cells, confirming the feasibility of therapeutic mitochondrial transplantation for tissue regeneration [91]. A powerful combination is fast fluorescence lifetime imaging microscopy (FLIM) with stimulated emission depletion (STED) nanoscopy [28]. FLIM measures the decay rate of fluorescence, an intrinsic property that changes with the local environment, allowing for real-time assessment of mitochondrial metabolic state (e.g., via NADH autofluorescence). STED provides super-resolution imaging, enabling the visualization of individual organelles within nanoscale TNT structures. Furthermore, flow cytometry offers a standardized, high-throughput method for quantifying transfer efficiency in large cell populations, as used in studies of chimeric cell therapy for Duchenne muscular dystrophy [92].

### 6.4. Artificial Intelligence (AI)-Driven Analysis

AI and machine learning are emerging as powerful tools for analyzing complex mitochondrial transfer datasets. Machine learning algorithms have been applied to develop prognostic indices based on mitochondrial function. The Mitochondrial Function and Cell Death Pattern Index (MPCDI), developed by integrating single-cell and bulk RNA sequencing data from multiple cancer datasets, successfully stratified hepatocellular carcinoma patients based on mitochondrial functional status and predicted their response to immunotherapy [93]. Similarly, a multi-omics, AI-derived prognostic signature based on mitochondria-related genes demonstrated robust predictive power for outcomes and therapeutic responses in lung adenocarcinoma [94].

AI is also helping to address technical challenges. Advanced filtering algorithms can now better distinguish cells with genuinely high mitochondrial content due to metabolic alterations from those with artifacts in single-cell studies, which is crucial as malignant cells often have higher baseline mitochondrial gene expression than healthy tissues [95]. AI platforms like scCamAge can even predict cellular age and aging-associated bioactivities, including mitochondrial dysfunction, from imaging data at single-cell resolution [96]. These computational advances, combined with precise quantification techniques, are clarifying the metabolic impact of transfer events, such as the increased ATP production and proliferation observed when breast cancer cells receive platelet-derived mitochondria [97].

## 7. Future Directions and Clinical Translation

### 7.1. Precision Biomarkers and Patient Stratification

The clinical translation of the MERCI methodology established specific implementation criteria for patient stratification. MERCI achieves 90% specificity and 80% precision at rank cutoffs between 20% and 50%, with a moderate sensitivity of 56.3% when analyzing patients with sufficient mitochondrial transfer activity [6]. The TMT score, derived from 17 genes implicated in nanotube formation and mitochondrial transfer, demonstrated a strong correlation with rapid tumor proliferation and poor patient survival across over 10,000 cancer samples [27].

Clinical validation studies have demonstrated that mitochondrial haplogroup T is associated with significant resistance to anti-PD-1-based immunotherapy, with patients carrying this haplogroup being 3.46 times less likely to respond to checkpoint therapy in melanoma [67]. Ultra-low-coverage whole-genome sequencing enables practical clinical implementation of haplogroup analysis for treatment selection.

Multiple features of circulating cell-free mtDNA, including copy number, mutations, and fragmentomics, provide predictive value for treatment response in patients with hepatocellular carcinoma receiving transarterial chemoembolization [65]. mtDNA copy number serves as a validated biomarker for guiding adjuvant chemotherapy decisions in colorectal cancer patients with mismatch repair deficiency [66].

Clinical implementation of these biomarkers requires standardized protocols and infrastructure development. TMT score calculation can be integrated into routine single-cell RNA sequencing workflows, with the 17-gene signature providing actionable cutoffs for patient stratification [6,27]. Mitochondrial haplogroup determination through ultra-low-coverage whole-genome sequencing offers a cost-effective approach for predicting checkpoint inhibitor resistance in melanoma patients [67]. For circulating biomarkers, cf-mtDNA analysis can be incorporated into existing liquid biopsy platforms, with copy number and mutation detection protocols already validated for hepatocellular carcinoma and colorectal cancer applications [65,66]. Implementation strategies must address workflow integration, result interpretation guidelines, and cost-effectiveness considerations to ensure widespread clinical adoption.

### 7.2. Combination Therapeutic Strategies

Bone marrow stromal cells establish talin2-dependent nanotubular connections with CD8+ T cells, enabling mitochondrial transfer that enhances T cell expansion, tumor infiltration, and resistance to exhaustion [7]. Mitochondria-boosted T cells demonstrate superior antitumor responses and enhanced compatibility with checkpoint inhibitor therapies. Importantly, mitochondria-enhanced T cells prone to exhaustion showed reduced PD-1, LAG3, and TIGIT expression levels, suggesting beneficial coupling with the PD-1:PD-L1 blockade.

Deleting the mitochondrial respiration negative regulator MCJ enhances CD8+ CAR-T cell efficacy by increasing mitochondrial membrane potential, maximal respiration, and spare respiratory capacity [24]. This approach significantly improves the cytotoxic function and persistence of CAR-T cells, particularly after multiple expansions with IL-2, addressing major limitations in CAR-T cell manufacturing and function.

Beyond these direct mitochondrial enhancement approaches, metabolic combination strategies offer additional synergistic opportunities with checkpoint inhibitors. Bezafibrate-mediated mitochondrial targeting enhances CD8+ T cell infiltration and can be combined with checkpoint inhibition in lung cancer models [40]. Similarly, targeting the TCA cycle through pyruvate and oxoglutarate dehydrogenase inhibition improves anti-PD-1 immunotherapy efficacy by modulating both metabolic flux and immune recognition pathways [53]. These metabolic interventions address the fundamental barriers that limit current immunotherapy effectiveness while enhancing the cellular fitness required for sustained antitumor responses.

### 7.3. Clinical Implementation Challenges

Current mitochondrial transfer rates of approximately 10% may limit clinical scalability, requiring optimization of transfer machinery and identification of “super donor” cell populations [7]. Standardized protocols for mitochondrial quality assessment, transfer efficiency monitoring, and therapeutic outcome evaluation require consistent clinical implementation.

Long-term safety monitoring must assess the persistence and integration of transferred mitochondria, particularly regarding potential immune responses to transferred organelles and their effects on cellular metabolism. The natural occurrence of mitochondrial transfer in physiological contexts provides foundational safety data. However, therapeutic enhancement requires a comprehensive evaluation of potential adverse effects.

Manufacturing costs, specialized infrastructure requirements, and complex quality control systems must be balanced to achieve therapeutic benefits. Cost-effectiveness models must incorporate personalized medical approaches, including biomarker testing expenses and potential healthcare utilization reduction through improved treatment outcomes.

Standardization of therapeutic mitochondrial transfer protocols presents critical challenges that must be addressed for clinical translation. Key gaps include establishing consistent criteria for donor cell selection, mitochondrial quality assessment, and transfer efficiency evaluation, as current protocols vary significantly between laboratories and cell populations. Manufacturing standardization requires addressing variability in transfer machinery expression, culture conditions, and expansion protocols that dramatically affect therapeutic outcomes. Additionally, the lack of standardized markers for assessing transferred mitochondrial integration, function, and persistence in recipient cells complicates treatment monitoring and dose optimization. Regulatory frameworks must be developed to ensure consistent quality control parameters across the entire therapeutic pipeline, from donor cell characterization to final product assessment, while standardized outcome measures and biomarker panels are essential for meaningful comparison of results across clinical trials.

Scaling therapeutic mitochondrial transfer approaches faces significant manufacturing and delivery challenges. The identification of “super donor” cell populations with enhanced transfer capabilities represents one approach to address the current 10% transfer efficiency limitation [7]. However, expanding production of these specialized cell populations requires standardized culture protocols and quality control measures that can be consistently implemented across multiple manufacturing sites. Additionally, the requirement for patient-specific or HLA-matched donor cells, as demonstrated with MSC-to-Treg transfer [72], complicates large-scale manufacturing and inventory management for widespread clinical application.

### 7.4. Addressing Current Knowledge Gaps: Experimental Priorities

Several key experiments are needed to address current knowledge gaps in mitochondrial transfer. To improve MERCI’s sensitivity beyond 56.3% [6], time-course single-cell RNA sequencing studies combined with real-time mitochondrial tracking could better capture dynamic transfer events. Investigating the selective transfer mechanisms identified in cancer cells [5,6] requires functional studies using fluorescently labeled healthy versus damaged mitochondria to directly visualize preferential exchange patterns.

The 10% transfer efficiency limitation [7] necessitates systematic screening of transfer-enhancing conditions, including optimization of Cofilin pathway modulation [4] and CX43 expression levels [21]. To address standardization challenges, multi-center studies comparing transfer protocols across different laboratories are essential for establishing reproducible clinical implementation guidelines. Finally, long-term safety studies tracking transferred mitochondrial persistence and integration are critical for addressing the tumorigenic risks highlighted with carcinoma-associated MSCs [23].

## 8. Conclusions

Mitochondrial transfer between cancer and T cells represents a fundamental mechanism of cellular communication that profoundly affects tumor–immune system interactions. This review demonstrates the dual nature of mitochondrial transfer as a mechanism exploited by cancer cells for immune evasion and a promising therapeutic avenue for enhancing immune function.

The development of the MERCI computational framework has enabled the systematic investigation of mitochondrial transfer at single-cell resolution, revealing reproducible transfer phenotypes associated with cytoskeleton remodeling, energy production, and poor clinical outcomes across multiple cancer types. Clinical validation has demonstrated that cancer cells can hijack functional mitochondria from infiltrating T cells while transferring dysfunctional organelles that promote immune suppression and treatment resistance.

The therapeutic applications of mitochondrial transfer have shown significant promise. Mitochondrial enhancement in bone marrow stromal cells improves T cell metabolic fitness, antitumor efficacy, and compatibility with checkpoint inhibition. CAR-T cell therapy substantially benefits from mitochondrial transfer, with enhanced survival and reduced manufacturing-related toxicity. The identification of mitochondrial haplogroups as predictive biomarkers of checkpoint inhibitor resistance provides a practical tool for treatment selection.

Clinical translation faces several key challenges, including the optimization of transfer efficiency, development of standardized manufacturing protocols, comprehensive safety assessments, and establishment of cost-effective implementation strategies. The current mitochondrial transfer rate of 10% requires improvement in clinical scalability, and regulatory frameworks must address novel aspects of organelle-based therapies.

Future research should focus on identifying the molecular mechanisms to enhance transfer efficiency, developing surrogate markers to avoid fluorescent labeling requirements, and establishing comprehensive quality control protocols. The integration of mitochondrial transfer modulation with existing immunotherapies represents a particularly promising approach for overcoming current therapeutic limitations and achieving improved patient outcomes in cancer treatment.

The emerging understanding of oxidative stress as a central regulator of mitochondrial transfer provides new therapeutic opportunities. Targeting the redox imbalance that drives pathological organelle trafficking—whether through antioxidant supplementation, ROS scavenging, or modulation of specific pathways like KEAP1-NRF2—represents a promising approach for redirecting mitochondrial transfer from immune-suppressive to immune-enhancing outcomes.

Understanding the complex interplay between mitochondrial transfer, metabolic rewiring, and immune function provides new perspectives on cancer biology and opens innovative avenues for therapeutic interventions. As our knowledge of these processes continues to expand through advanced analytical technologies, modulation of mitochondrial transfer may become a cornerstone of next-generation cancer immunotherapy.

## Figures and Tables

**Table 1:** Comparative features of mitochondrial transfer mechanisms.

Feature	Tunneling Nanotubes (TNTs)	Extracellular Vesicles (EVs)	Direct Cell–Cell Contact	References
Structural Basis	F-actin-based membrane tubes creating continuous cytoplasmic channels	Membrane-bound vesicles packaging mitochondrial components	Gap junction channels formed by Connexin proteins	[9,18,21]
Key Regulatory Proteins	Cofilin (actin-depolymerizing factor), Miro1 (adaptor protein)	P2X7 receptor (ATP-gated ion channel)	Connexin 43 (CX43)	[4,20,21,22]
Formation Triggers	Oxidative stress, microenvironmental stress	Sustained ATP activation, cellular stress	Oxidative stress in recipient cells	[4,20,21]
Cargo Transfer	Whole intact organelles	Mitochondrial proteins, metabolites, mtDNA	Whole organelles and small molecules	[9,18,21]
Therapeutic Targeting	Cofilin pathway inhibition, optical manipulation	P2X7 receptor modulation, engineered vesicles	CX43 expression modulation	[4,9,20,21,29]
Detection Methods	FLIM-STED nanoscopy, real-time imaging	Liquid biopsy platforms, tissue-specific analysis	Single-cell RNA sequencing	[18,28,30]
Cancer Applications	Adenoid cystic carcinoma, prostate cancer	Broad cancer applications	Melanoma, hematologic malignancies	[14,16,18,22,30]
Functional Outcomes	Cell fusion, metabolic rescue	Long-range signaling, biomarker potential	Stemness maintenance, metabolic support	[14,18,30]

**Table 2:** Clinical biomarkers for mitochondrial transfer assessment.

Biomarker Category	Specific Marker	Clinical Application	Performance Metrics	Cancer Types	Clinical Status	References
Computational Signatures	TMT score (17-gene signature)	Mitochondrial transfer quantification and prognosis	90% specificity 80% precision	Pan-cancer	Clinically Validated	[6,27]
Mitochondrial Genetics	Haplogroup T (HG-T)	Anti-PD-1 resistance prediction	3.46-fold increased resistance	Melanoma	Clinically Validated	[67]
Circulating Biomarkers	cf-mtDNA copy number, cf-mtDNA mutations	Treatment response prediction, Treatment response monitoring	Predictive for TACE response	Hepatocellular carcinoma	In Clinical Use	[65]
	cf-mtDNA fragmentomics	Treatment response assessment	Fragment pattern analysis	Hepatocellular carcinoma	Preclinical/Research	[65]
Tissue Biomarkers	mtDNA copy number	Adjuvant therapy guidance	Guides stages II/III treatment	Colorectal cancer (MMR-deficient)	In Clinical Use	[66]
Protein Biomarkers	LCLAT1 expression	Prognosis prediction, Immunity prediction	Multi-parameter assessment	Hepatocellular carcinoma	Clinically Validated	[69]
	Proteomic signatures	Immunotherapy, response prediction	High-accuracy prediction	Melanoma	Clinically Validated	[68]

**Table 3:** Therapeutic strategies targeting mitochondrial transfer.

Strategy Category	Specific Approach	Mechanism of Action	Target Population	Key Efficacy Data	Development Stage	References
Enhancement Therapies	BMSC-mediated transfer	Talin2-dependent nanotubular connections	CD8+ T cells, CAR-T cells	Enhanced expansion, reduced exhaustion	Preclinical/Research	[7]
	MCJ deletion, MCJ silencing	Deletion of mitochondrial Complex I negative regulator MCJ	CAR-T cells, exhausted T cells	Enhanced mitochondrial respiration improved CAR-T survival and efficacy	Preclinical/Research	[24]
	Selenium nanoparticle activation	Enhances MSC transfer capacity via selenoproteins	Mesenchymal stem cells	Enhanced transfer efficiency	Preclinical/Research	[63]
	Direct mitochondrial transplantation	Cell-free healthy organelle replacement	Multiple tissue types	Promising in animal models	Early Clinical	[64]
Metabolic Modulators	Nicotinamide riboside (NR)	NAD+ precursor mitochondrial restoration	Exhausted T cells	Enhanced mitochondrial fitness	Early Clinical (Phase I/II)	[38]
	Myoglobin expression	Improved oxygen utilization in T cells	Engineered T cells	Increased ATP, enhanced function	Preclinical/Research	[41]
	P4HA1 targeting	Enhances mitochondrial function	CD8+ T cells	Enhanced antitumor immunity	Preclinical/Research	[39]
	Bezafibrate	PPAR agonist mitochondrial targeting	CD8+ T cells	Improved tumor infiltration	Preclinical/Research	[40]
Delivery Systems	Mitophagy agonist nanofactories	Hydrogel co-delivery with CAR-T cells	CAR-T cells in solid tumors	Enhanced memory formation	Preclinical/Research	[42]
	STING-activating nanofactories	Regulates mitochondrial dysfunction	Exhausted T cells	Restored immune activation	Preclinical/Research	[43]
Inhibition Strategies	TNT formation inhibitors	Cofilin pathway targeting	Cancer-immune interactions	Reduced pathological transfer	Preclinical/Research	[4,17]
	EV release/uptake inhibitors	Targeting P2X7 receptor, Targeting MDV pathways	Detrimental transfer contexts	Blockade of pathological transfer	Preclinical/Research	[20,29]
	Optical manipulation	Near-infrared control of TNTs	Precision cell targeting	Selective transfer inhibition	Preclinical/Research	[9]

## Abbreviations

The following abbreviations are used in this manuscript:

AI	artificial intelligence
BMSC	bone marrow-derived mesenchymal stromal cell
CAF	cancer-associated fibroblast
CAR-T	chimeric antigen receptor T
cf-mtDNA	circulating cell-free mitochondrial DNA
CLL	chronic lymphocytic leukemia
EV	extracellular vesicle
KEAP1	Kelch-like ECH-associated protein 1
LCLAT1	lysocardiolipin acyltransferase 1
MDV	mitochondria-derived vesicle
MERCI	mitochondrial-enabled reconstruction of cellular interaction
MHC	major histocompatibility complex
mitoEV	mitochondria-containing extracellular vesicle
MCJ	methylation-controlled J
MSC	mesenchymal stem cell
mtDNA	mitochondrial DNA
NR	nicotinamide riboside
NRF2	nuclear factor erythroid 2-related factor 2
OXPHOS	oxidative phosphorylation
P2X7	Purinergic Receptor P2X 7
P4HA1	prolyl 4-hydroxylase α1
REC	rapidly expanding clone
ROS	reactive oxygen species
TCA	tricarboxylic acid
TIL	tumor-infiltrating lymphocyte
TME	tumor microenvironment
TMT	tumor mitochondrial transfer
TNT	tunneling nanotube
TRM	tissue-resident memory
